# Strength of density feedback in census data increases from slow to fast life histories

**DOI:** 10.1002/ece3.298

**Published:** 2012-07-12

**Authors:** Salvador Herrando-Pérez, Steven Delean, Barry W Brook, Corey J A Bradshaw

**Affiliations:** 1 The Environment Institute and School of Earth and Environmental Sciences, University of AdelaideSouth Australia, 5005, Australia; 2 South Australian Research and Development InstituteP.O. Box 120, Henley Beach, South Australia, 5022, Australia

**Keywords:** Age at first reproduction, body size, density dependence, fertility, longevity, population dynamics

## Abstract

Life-history theory predicts an increasing rate of population growth among species arranged along a continuum from slow to fast life histories. We examine the effects of this continuum on density-feedback strength estimated using long-term census data from >700 vertebrates, invertebrates, and plants. Four life-history traits (*Age at first reproduction, Body size, Fertility, Longevity*) were related statistically to Gompertz strength of density feedback using generalized linear mixed-effects models and multi-model inference. Life-history traits alone explained 10 to 30% of the variation in strength across species (after controlling for time-series length and phylogenetic nonindependence). Effect sizes were largest for body size in mammals and longevity in birds, and density feedback was consistently stronger for smaller-bodied and shorter-lived species. Overcompensatory density feedback (strength <−1) occurred in 20% of species, predominantly at the fast end of the life-history continuum, implying relatively high population variability. These results support the idea that life history leaves an evolutionary signal in long-term population trends as inferred from census data. Where there is a lack of detailed demographic data, broad life-history information can inform management and conservation decisions about rebound capacity from low numbers, and propensity to fluctuate, of arrays of species in areas planned for development, harvesting, protection, and population recovery.

## Introduction

Density dependence (Smith 1935; Allee 1941) represents a causal relationship between population size (predictor) and a demographic rate (response), that is, a “density feedback”. Such relationship can be “compensatory” or “depensatory” if population growth, survival, and/or fertility rates decrease or increase with population boom, respectively (Herrando-Pérez et al. 2012a). Statistical support for those feedbacks indicates that demographic rates are shaped by social and trophic interactions such as competition, cooperation, disease, parasitism, or predation, because the intensity of these ecological mechanisms varies with population size (Herrando-Pérez et al. 2012b). In single-species population models that quantify density feedback (Brook and Bradshaw 2006; Eberhardt et al. 2008), it has been suggested that cross-taxa patterns of population dynamics can be predicted from information on life-history traits by arranging species along a continuum from “slow” to “fast” life histories (Saether et al. 2002). This continuum had been thoroughly investigated in the 1980s in homeotherms (Saether 1987; Galliard et al. 1989; Read and Harvey 1989) – the prediction being that fast taxa should be capable of growing to larger population sizes at much higher rates than slow taxa via the former's shorter gestation, shorter intervals between reproductive bouts, earlier maturity, smaller adult size, shorter life, shorter lactation, smaller and more prolific offspring, and more litters per year, regardless of whether one controls for body size (Stearns 1983; Saether et al. 1996). In support of such predictions, changes in population growth rate (sensitivities) arise mainly from variability in reproductive rates in fast species and in survival rates for slow species of birds (Saether and Bakke 2000), mammals (Heppell et al. 2000; Oli and Dobson 2003; Oli et al. 2005), fish (Cortés 2002), insects (Blackburn 1991), and plants (Franco and Silvertown 2004). It is therefore reasonable to postulate that the position of a species along this continuum could also reflect the propensity of population growth rates to vary in response to social/trophic interactions among individuals, as inferred from metrics of density feedback.

Evidence for density feedback increases with longevity based on census data from bird species (Holyoak and Baillie 1996), and species with slow life histories (longer generation times, larger body size, smaller litters) experience more demographic stability when compensatory density feedbacks operate than fast species with recruitment-driven dynamics (Saether et al. 2002). The only two studies that have investigated this matter over broad taxonomic groups have focused on the shape (i.e., nonlinearity) of density feedback and provided conflicting results. First, Fowler (1981) formalized the links between convex (compensatory feedback strongest at high numbers) and concave (compensatory feedback strongest at low numbers) density feedback with the life histories of (large-bodied) mammals and (small-bodied) insects, respectively. He later showed that the inflection point of animal population growth curves declined with accelerating growth per generation (hence from slow to fast species), irrespective of body size (Fowler 1988). Conversely, Sibly et al. (2005) claimed an unprecedented ubiquity of concave density feedback across mammals, birds, bony fish, and insects by applying a modified, curve-fitting form of the theta-logistic equation (Gilpin and Ayala 1973), with a change from concave to convex density feedback from large to small body-sized mammals. This article was repeatedly challenged immediately after publication (Doncaster 2006; Getz and Lloyd-Smith 2006; Ross 2006), and its conclusions soundly refuted due to fundamental flaws in the model-fitting approach employed (Doncaster 2008; Polansky et al. 2009; Ross 2009; Clark et al. 2010).

However controversial, the conclusions from these kinds of studies are of immediate relevance to conservation and management, because strength and shape of density feedback can dictate the (theoretical) capacity of a population to recover from declines following natural perturbations and/or harvest, thus exerting a strong influence on predictions of population extinction and viability (Henle et al. 2004; Sabo et al. 2004), and harvesting quotas (Boyce et al. 1999; Rose et al. 2001). So, while Fowler (1981) suggested that large mammals should be harvested at population sizes close to carrying capacity (where their productivity is expected to peak given convex density feedback), Sibly et al. (2005) stated that population growth rates could be overestimated if convex density feedback is assumed from life-history data (e.g., body size), with potentially serious implications for harvesting and management.

Here, we quantify strength of density feedback across several hundred taxa (vertebrates, invertebrates, plants), and determine effect sizes of and how much variance can be explained by four life-history traits (age at first reproduction, body size, fertility, longevity) which capture the slow–fast continuum. We hypothesize that the strength of compensatory density feedbacks increases along this continuum, that is, from low to high fertility, extended to short longevity, late to early age at first reproduction, and large to small body size.

## Materials and Methods

### Data

We used the data set of Brook and Bradshaw (2006) and Brook et al. (2006). In summary, these data consist of one census of population abundance and one estimate of four life-history traits for each of 1198 species (603 insects, 225 birds, 152 mammals, 115 fish, 36 aquatic invertebrates, 30 plants, 27 amphibians, and 10 reptiles), and feature >10 population counts per census (median = 20, with 95% percentile range of [10–65]). We deemed an annual time step appropriate to estimate population turnover because most species' census data were collected from temperate regions, hence they generally experience pronounced annual seasonality in reproductive events and survival.

We obtained species-specific life-history traits from independent sources (e.g., http://www.demogr.mpg.de/longevityrecords, http://www.bto.org, or genomics.senescence.info) for each of the 1198 species: (*i*) average age at first reproduction (months), (*ii*) maximum body size (length in mm), (*iii*) fertility (number of young per year), and (*iv*) longevity (maximum age attained in the wild in months) (Brook et al. 2006). These traits suffice to capture the principal features of the slow–fast continuum in mammals and birds (Galliard et al. 1989), and fall within the group of traits originally used to define this continuum (Stearns 1983). We explored correlations between traits representing gradients of life history across taxa through principal-component analysis (Jolliffe 2004).

### Strength of density feedback

Following Brook and Bradshaw (2006), we ranked evidence for Ricker-logistic and Gompertz population growth (density feedback present) against models of random walk and exponential growth (density feedback absent) by means of Akaike's information criterion corrected for finite sample size (AICc, Sugiura 1978). AIC_*c*_ and the Bayesian information criterion (BIC; Schwarz 1978), had approximately equivalent penalty terms for the median time-series length in our samples and thus produced qualitatively similar results.

For those time series supported for Gompertz growth, and having similar support for both Gompertz and Ricker-logistic growth (ΔAIC_*c*_ <4), we collated the estimates of strength of compensatory density feedback from the Gompertz equation (Medawar 1940; Nelder 1961), that is, the slope of the relationship of *r* [= log_*e*_(*N*_*t*+1_/*N*_*t*_)] versus population size on a log scale:


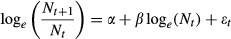


where *N*_*t*_ = population size at time *t*, *α* = intercept, *β* = strength of density feedback, and *ε*_*t*_ = Gaussian random variable with a mean of zero and a variance *σ*^2^ reflecting stochastic variability in *r*. This model (*i*) is measured on a proportional scale and so characterizes the multiplicative nature of demographic rates (Bjørnstad et al. 1995), (*ii*) clearly informs the magnitude of the compensatory response of demographic rates to changes in population size relative to nonlinear models (Doncaster 2006), and (*iii*) slopes above and below –1 represent the threshold between expected stable and chaotic dynamics, respectively, and so provide a simple metric with which to assess population variability (Varley et al. 1973; Doncaster 2008) **–** slopes <–1 imply that the proportional number of survivors over any time step of a census decreases by >100% for a one-order-magnitude increase in population size. Furthermore, the Gompertz model has performed robustly in describing the general dynamics of populations over a wide range of body sizes (Saitoh et al. 1999; Wang et al. 2002, 2009; White et al. 2007; Seavy et al. 2009; Pasinelli et al. 2011) is present in multi-model inference scenarios where competing models are contrasted (Saitoh et al. 1997; Zeng et al. 1998; Fryxell et al. 2005; Chamaillé-Jammes et al. 2008; McMahon et al. 2009), is the top-ranked model in meta-analyses of hundreds of species in which various alternatives have also been evaluated (Brook and Bradshaw 2006), and has been a model used in theoretical development about density feedback (Dennis et al. 2006). We avoided fitting the fully parameterized theta-logistic model (see Introduction), or other highly parameterized analogs (e.g., hyperbolic growth). Yet, we also did all analyses using the Ricker-logistic strength of density feedback as response. In our study, we make no claim about the regulation of populations, because moderate compensatory density feedback is only one requirement for population regulation (Herrando-Pérez et al. 2012b).

### Model set

With Gompertz strength of compensatory density feedback as the common response, our model set included 10 models with the following predictors (Table 1): (*i*) four models with each single life-history trait alone, (*ii*) four models with fertility and one of the other traits, (*iii*) the intercept-only (null) model with no fitted predictor terms, and (*iv*) one model controlling for sample size (length of the time series) only. The ratio of fertility to age at first reproduction has been proposed as a metric of the slow–fast continuum in mammals (Oli and Dobson 2003) and was also included in the model set. Fertility is an obvious proxy for reproductive rates, while body size, longevity, and age at first reproduction are directly related to survival (Galliard et al. 1989; Saether et al. 1996), so our models incorporated life-history selection for those demographic rates. As the length of the time series affects the detection probability of density feedback (Brook and Bradshaw 2006), we included it in all models encompassing life-history traits and then calculated the variance explained by life-history parameters alone. A priori, we explored pair-wise correlations between life-history traits and did not include strongly co-linear traits in our model contrasts (except when controlling for body size [see below]); and we dispensed with any interaction terms due to the difficulty of their interpretation in this context, thus avoiding over-parameterizing models. Finally, we did not use the principal components of our PCA (principal component analysis) analyses (see above) as predictors in our models because we were not interested in (potentially) maximizing model goodness of fit, but mainly in teasing apart the relative fixed effects of single life-history traits.

**Table 1:** Model sets including predictors of variation in strength of compensatory density feedback across taxa (fitted by GLMM using as random factor CL = taxonomic *Class*), and bird and mammal species (fitted by GLM, no phylogenetic random effect). Life-history traits included *Age*, Age at first reproduction (months); *Body*, Body size (millimeters); *Fert*, Fertility (number of young per year); *Long*, Longevity (maximum age attained in the wild, months). Control variables were included in all models (except the null), namely *q* = length of time series, *G* = number of generations monitored (*q/Age*), and body size

*q* or *G*	Body size
Control variables q	
1 + (1 | CL)	
*q* + (1 | CL)	1 + (1 | CL)
*q* + *Body* + (1 | CL)	*q* + (1 | CL)
*q* + *Age* + (1 | CL)	*q* + *Body* + (1 | CL)
*q* + *Fert* + (1 | CL)	*q* + *Body* + *Age* + (1 | CL)
*q* + *Long* + (1 | CL)	*q* + *Body* + *Fert* + (1 | CL)
*q* + *Body* + *Fert* + (1 | CL)	*q* + *Body* + *Long* + (1 | CL)
*q* + *Age* + *Fert* + (1 | CL)	*q* + *Body* + *Age* + *Fert* + (1 | CL)
*q* + *Long* + *Fert* + (1 | CL)	*q* + *Body* + *Long* + *Fert* + (1 | CL)
*q* + *Fert*/*Age* + (1 | CL)	*q* + *Body* + *Fert*/*Age* + (1 | CL)
Control variables G	
1 + (1 | CL)	
*G* + (1 | CL)	1 + (1 | CL)
*G* + *Body* + (1 | CL)	*G* + (1 | CL)
*G* + *Fert* + (1 | CL)	*G* + *Body* + (1 | CL)
*G* + *Long* + (1 | CL)	*G* + *Body* + *Fert* + (1 | CL)
*G* + *Body* + *Fert* + (1 | CL)	*G* + *Body* + *Long* + (1 | CL)
*G* + *Long* + *Fert* + (1 | CL)	*G* + *Body* + *Long* + *Fert* + (1 | CL)

### Model fitting

We fitted all models using generalized linear mixed-effects models (GLMM; Breslow and Clayton 1993). Model assumptions were met using a Gaussian variance function after a square-root transformation of density-feedback strengths, such transformation being supported by a likelihood-based test of Box and Cox (1964). Covariance between life-history traits should be incorporated in cross-taxa comparisons of demographic and evolutionary responses (Felsenstein 1985), and can be accounted for by allowing different intercepts for species grouped by higher taxonomic levels (Blackburn and Duncan 2001). We did so by including the Linnaean taxonomic level of *Class* as a random effect in our GLMMs (Table 1). We discarded nested random factors by *Family* and *Order* due to insufficient replication over half of the families and orders; we also replicated our analyses for birds and mammals (see below) separately using generalized linear models (GLM).

We quantified relative support for models in a set by means of the BIC (Schwarz 1978) because BIC favors more parsimonious models than AIC when sample sizes are large (∼50 to 300 estimates of strength in any of our model contrasts) and we wanted to distinguish main from tapering effects (Burnham and Anderson 2002; Link and Barker 2006). Nevertheless, both BIC and AIC yielded nearly identical model support and the same biological conclusions emerged. Exploratory analyses confirmed that, within each of 12 (“redundant studies” hereafter) of the 204 peer-reviewed data sources, individual species' time series had equal time-series length, and life-history traits had equal or similar values. Such redundant information was bound to overwhelm model fitting since it affected 613 species (∼60% of the data set, of which 519 were insects, for instance, multi-species monitoring of aphids and moths at single field stations). To avoid this, we separated “redundant species” from the remaining (nonredundant) “core species.” Of the 772 times series supported for Gompertz growth, 326 belonged to “core species” and 446 were from “redundant species.” After accounting for data redundancy, we could fit our models robustly to all taxa, mammals, and birds. We calculated model ranking and relative fixed effects on 100 data subsets, each consisting of one bootstrapped sample of all core species and one randomly sampled species from each of the redundant studies – that is, 100 contrasts of the same model set, each time on a different bootstrapped sample. We measured relative model support across the set by the medians and 95 percentile confidence intervals of BIC metrics (ΔBIC, model probabilities, deviances) over the 100 bootstrapped samples. Furthermore, we used model averaging (Burnham and Anderson 2002) to estimate the coefficients of the fixed effects for each life-history trait on strength of density feedback. Thus, we summed model probabilities for each model containing a given life-history trait weighted by its effect size as a measure of across-model effect size. To confirm that effect sizes were comparable among life-history traits of different range, we assessed them with and without a post hoc standardization (trait × SD [response]/SD [trait]).

### Complementary analyses

To avoid the confounding effects of measuring error, authors either set stringent criteria for data selection (Knape and Valpine 2011), or use state-space models (Dennis et al. 2006; Knape 2008; Ives et al. 2010), which themselves are not, however, exempt of caveats (Knape 2008) and add further model complexity to cross-taxa comparisons. Therefore, we decided to replicate all analyses for (*i*) the entire data set, (*ii*) a subset of “high-quality” time series featuring stationarity, no temporal trending, few missing values, no outliers, and length of counts of >14 time steps (these criteria are fully explained in Table S2), and (*iii*) simulated time series from the observed Gompertz parameters with incorporation of 5%, 10%, and 15% of measurement error (we present this simulation in the Supporting Information [Table S7]).

We did not have access to estimates of each species' “generation time” as used elsewhere to relate single-species population models to life history, for example, (Saether et al. 2005, 2004). As body size correlates with generation time and intrinsic growth rates (Peters 1983), and needs to be accounted for when studying the slow–fast life-history continuum (Stearns 1983; Galliard et al. 1989; Jeschke and Kokko 2009), we controlled for allometric relationships among species by redoing all analyses with a model set including body size in all models, then compared relative effect sizes of each life-history trait in model sets with and without the control for body size. As a further control for generation time, we repeated all analyses using the number of generations monitored in each census (*G* = time-series length/age at first reproduction); this model set had seven candidate models after removing those including age at first reproduction (Table 1). We did all data analyses in R v2.14 (R Development Core Team 2011), and gave sample sizes for every model contrast in Table S1.

## Results

### Magnitude of density feedback across taxa

Median model probabilities (with 95% percentile ranges) for all species were 0.38 (0.07–0.97) for Gompertz, 0.23 (0.02–0.75) for Ricker-logistic, 0.16 (<0.01–0.67) for random walk, and 0.05 (<0.01–0.30) for exponential population growth. We found support for either of the two density-dependent models in 865 censuses, with total median evidence of 0.78 (0.14–1.00) (pooled model probability for Gompertz and Ricker-logistic models). Overall, the median probability of a population to show evidence for compensatory density feedback was 3.5 times that of not showing so. A total of 772 times series (64%, all data) (and 583 or 73% of the high-quality time series) were supported for Gompertz growth. We employed those subsets of 772 and 583 censuses in further analyses (Table S1).

Median strength of density feedback was –0.7 (–1.4 to –0.2), so increases in population size by a factor of ∼3 (i.e., one order of magnitude on a natural logarithm scale) caused a median 70% reduction in population growth rates. In order of strength by major taxonomic groups, plants were highest at –0.9 (–1.3 to –0.6), followed by herpetiles at –0.9 (–1.4 to –0.5), aquatic invertebrates at –0.8 (–1.5 to –0.2), insects at –0.8 (–1.4 to –0.3), fish at –0.7 (–1.3 to –0.2), birds at –0.6 (–1.3 to –0.2), and mammals at –0.4 (–1.4 to –0.1). We found similar magnitude and across-taxa ordering of strength of density feedback for the high-quality time series.

Overcompensatory density feedback (strength <−1) occurred in ∼20% of the censuses (all and high-quality time series), being relatively common among some of the small-bodied species of amphibians (seven species, 40% of amphibians), insects (96, 22%), fish (11, 21%), mammals (16, 18%), and birds (15, 10%). For instance, the five strongest density feedbacks were for the small tortoiseshell nymphalid *Aglais urticae* (–1.9 ± 0.4 SE), the common shrew *Sorex araneus* (–1.8 ± 0.2 SE), the leaf miner agromyzid *Chromatomyia suikazurae* (–1.7 ± 0.5 SE), the red crossbill finch *Loxia curvirostra* (–1.6 ± 0.3 SE), and the oak aphid *Tuberculatus annulatus* (–1.6 ± 0.3 SE).

### Predicting strength of density feedback from life history

The variation in strength of density feedback explained by life-history traits alone was 8 to 25% across all species, 28 to 34% for mammals, and 10 to 17% for birds over models controlling for census length (Table 2) and number of generations monitored (Table S4). Top-ranked models included longevity for all species and for just birds, and body size in mammals (Tables 2 and S4). Length of time series alone explained up to 21% (all species), 17% (mammals), and 10% (birds) of the variation in strength (Table 2), while the explanatory capacity was much lower for the number of generations monitored, that is, 0.1% (all species), 9% (mammals), and 2% (birds) (Table S4). Moreover, models including life-history traits had between 20 and >1000 times higher statistical support than models including only either of the two control variables (evidence ratios given in Tables 2 and S4). Importantly, the same model rankings and similar explained deviances occurred in simulated time series after incorporation of 5% measurement error in all taxa and birds, and up to 10% in mammals (Table S7).

**Table 2:** Bayesian information criterion (BIC) support for the top-ranked models^a^ relating life history to strength of density feedback through GLMM for all taxa (aquatic invertebrates, amphibians, birds, fish, insects, mammals, plants, reptiles), and GLM for the subsets of mammal and bird species. *w*BIC,%DE and Δ%DE are medians (in bold) from 100 bootstrapped samples (95% percentile range)^b^. Models included time-series length (*q*), and body size (*Body*) as controls. We show effect sizes in Figure 1, model sets in Table 1, and sample sizes in Table S1

Data set	Control variable	Top-ranked model per model set	*w*BIC	%DE	Δ%DE	ER	% Top-rank consistency
All taxa	*q*	*Strength ˜ q+Long*	**0.57** (0.00–1.00)	**30.7** (22.2–42.2)	**9.5** (5.2–15.4)	**>1000**	**54** (19)
All taxa	*q*,*Body*	*Strength ˜ q+Body+Long*	**0.64** (0.01–0.99)	**29.0** (21.1–40.4)	**8.2** (3.4–13.8)	**>1000**	**63** (16)
Mammals	*q*	*Strength ˜ q+Body*	**0.79** (0.07–0.90)	**45.2** (31.6–61.8)	**28.9** (12.2–50.5)	**>1000**	**85** (11)
Mammals	*q*,*Body*	*Strength ˜ q+Body*	**0.59** (0.09–0.71)	**45.2** (31.6–61.8)	**28.3** (12.2–50.5)	**>1000**	**80** (18)
Birds	*q*	*Strength ˜ q+Long*	**0.60** (0.04–0.91)	**19.4** (8.8–34.4)	**10.0** (2.4–20.4)	**200**	**75** (20)
Birds	*q*,*Body*	*Strength ˜ q+Body+Long*	**0.29** (0.02–0.88)	**21.1** (9.2–35.5)	**11.0** (3.2–21.3)	**20**	**43** (42)

a Model sets: One single response (*Strength* of compensatory density feedback), *and* 1 or 2 life-history predictors (*Age* = Age at first reproduction [months]; *Body*, Body size [mm]; *Fert*, Fertility [number of young per year]; and *Long*, Longevity [maximum age attained in the wild, months]).

b BIC metrics: *w*BIC, BIC Model probabilities given each data and model set; %DE, % Deviance in *Strength* explained by each model within each model set; Δ%DE, % Deviance in *Strength* explained by each model minus% Deviance in *Strength* explained by the model including only *q* (i.e., Deviance in *Strength* explained by life history conditional on *q*); ER, Evidence ratio of the top-ranked model *w*BIC compared to *q*-only model *w*BIC for each model set (i.e., times support for top-ranked model equating life-history traits was larger than for the only *q* model); and %Top-ranked consistency, times a model was top-ranked over the 100 bootstrapped samples (times each model was not the top-ranked model yet received considerable support [ΔBIC < 4])

**Figure 1:**
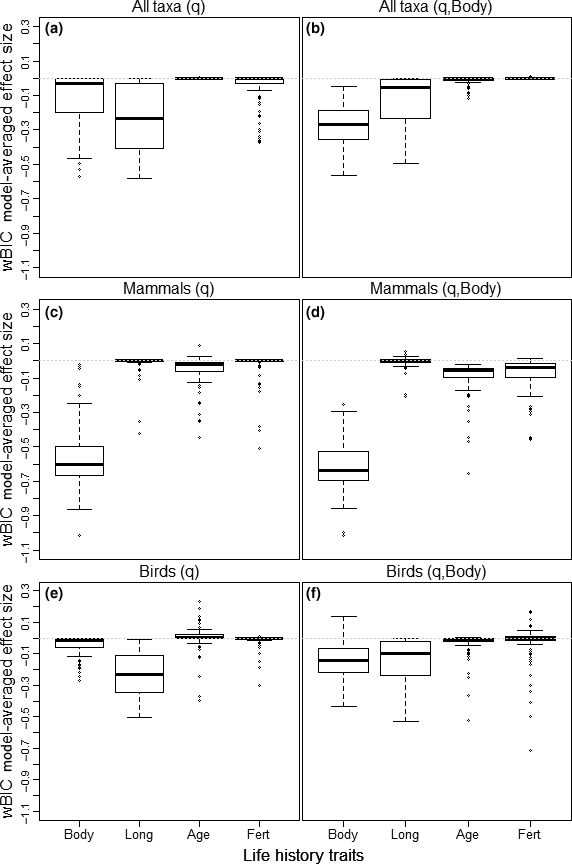
Standardized BIC-weighted effect sizes for four life-history traits (body size, longevity, age at first reproduction, fertility) as predictors of variation in strength of density feedback (response) for all major taxa (a,b: aquatic invertebrates, amphibians, birds, fish, insects, mammals, plants, reptiles), and the subsets of mammal (c,d) and bird (e,f) species. Left panels (a,c,e) come from a model set controlling for census length (*q*), and right panels (b,d,f) from a model set controlling for *q* and body size. Bold lines represent *w*BIC medians as obtained from 100 bootstrapped samples. Fits were obtained using GLMM which accounted for phylogenetic nonindependence at the Linnean taxonomical level of *Class*, and GLM for mammals and birds. We show model sets in Table 1, BIC metrics in Table 2, and sample sizes in Table S1.

BIC model-averaged fixed effects were largest for longevity (all species and birds) and body size (mammals) (Figs. 1 and S1, a,c,e). All effect sizes were negative (Figs. 1 and S1, a,c,e). Thus, the strength of density feedback increased from long- to short-lived life history across all species and birds, and from large- to small-bodied mammals. Age at first reproduction and fertility effects scored relatively small model-averaged effect sizes (Figs. 1 and S1, a,c,e). The trends above prevailed when we controlled for body size (Figs. 1, and S1, panels b,d,f), for the high-quality data set (Tables S5 and S6; Figs. S2 and S3), and using the Ricker-logistic strength of density feedback as response in all model contrasts.

### Life-history gradients

**Figure 2:**
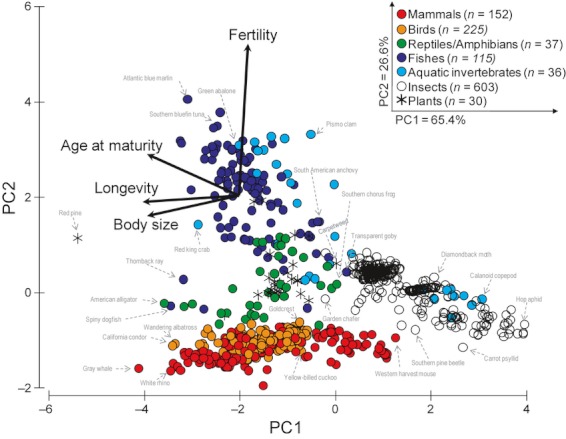
Correlation-based PCA of species based on log-transformed values of four life-history traits: age at first reproduction (months), body size (mm), fertility (number of young per year), and longevity (maximum age attained in the wild in months). Arrows represent principal coefficients assigned to each life-history trait, and indicate direction of increasing magnitude for any given life-history trait. Percentage correlation structure explained by each axis and number of species within broad taxa are given in legend. Examples of some species' common names are overlain in light gray and their position indicated with dashed arrows along life-history gradients. Legend shows samples sizes, also summarized in Table S1.

The first two principal-component axes (Fig. 2) explained 92% of the correlation structure among life-history traits across all species. The PC1 gradient separated insects from all other taxa, indicating (from right to left) increasing age at first reproduction, body size, and longevity, with considerable variation in life history within major vertebrate groups and aquatic invertebrates. The PC1 gradient is representative of the slow–fast continuum, and accounts for 65.4% of life-history correlations. The PC2 gradient separated homeothermic vertebrates (birds and mammals) from poikilothermic vertebrates (fish, reptiles, and amphibians), plants, and most insects and aquatic invertebrates (Fig. 2). This second gradient mainly represented (from bottom to top) increasing fertility, especially in aquatic species with broadcast-spawning bony fish (e.g., Atlantic blue marlin *Makaira nigricans*, southern bluefin tuna *Thunus maccoyii*) and megamolluscs (e.g., green abalone *Haliotis fulgens*, pismo clam *Tivela stultorum*). Fish species showed the largest relative life-history variation (i.e., spread in PCA space) within major taxa. This PC2 gradient is representative of reproductive output, explaining 26.6% of life-history correlations. Thus, given a species' position along the slow–fast continuum (PC1), relatively disparate interspecific reproduction output (PC2) occurred in all taxa except birds and mammals. Considering species within the best represented taxa, fertility correlated negatively with age at first reproduction, body size, and longevity in mammals and birds, and positively in fish and insects. The former represented overall increase in fertility from small- to large-bodied poikilotherms, and from large- to small-bodied homeotherms. We observed a similar gradient of life history for the high-quality data subset. Given the results of the PCA, our data provide a biological meaningful ranking of species along the slow–fast continuum of life histories.

## Discussion

Our results support the hypothesis that the position of a species along the slow–fast continuum of life histories imprints an evolutionary signal in population trajectories apparently shaped by density feedback across several hundred species of invertebrates, vertebrates, and plants. Previous studies, using different statistical approaches and plagued by violated assumptions (see below), provided simple correlations between life history and metrics of density feedback (Saether et al. 2002; Sibly et al. 2005; Saether et al. 2005), inflection points (Fowler 1981, 1988; Sibly et al. 2005), return to carrying capacity (Sibly et al. 2007), or sensitivities (Saether and Bakke 2000; Heppell et al. 2000; Oli and Dobson 2003; Franco and Silvertown 2004). We thus give the first robust quantitative assessment of the correlation between density feedback and life history, simultaneously including controls for taxonomy, allometry, prediction for several traits ecologically and evolutionarily related to fertility and survival, and a quantification of relative effect sizes.

Matching metabolic expectations, allometry (i.e., body size) accounts for most of the explained variation in strength of density feedback across all taxa, especially in mammals (Duncan et al. 2007; Sibly and Brown 2007). Indeed, fast life histories characterize species with relatively low per capita biomass production (Ernest et al. 2003), high intrinsic rates of increase, and high population densities and larger energy investments in reproduction relative to body maintenance (Fenchel 1974; Blueweiss et al. 1978; Brown et al. 2004), as a result, stronger compensatory density feedbacks are expected. After accounting for allometry, we found that the effect sizes of other life-history traits on the strength of compensatory density feedbacks remain low in mammals, but longevity remains a good predictor for birds. Mammals show the widest range of body sizes among living vertebrates; however, body size in most birds is constrained by flight, and longevity instead seems to be selected for along the slow–fast continuum for this group (Galliard et al. 1989). Furthermore, Gompertz strengths <–1 are indicative of overcompensatory density feedback, which can result in populations overshooting carrying capacity and undergoing chaotic fluctuations (Varley et al. 1973). We predicted such overcompensatory feedbacks in ∼20% of all taxa and mammals, and ∼10% of birds, mainly at the fast end of the life-history continuum. This implies more population variability in the long term for fast species, as has indeed been shown for birds (Saether et al. 2002, 2004; Saether and Engen 2002) and mammals (Sinclair 1996; Erb et al. 2001; Fagan et al. 2001).

We used four life-history traits (age at first reproduction, body size, fertility, longevity) to represent the slow–fast continuum. However, life-history signaling in census data might be even stronger than detected here if other traits (particularly size of individual offspring and frequency of reproductive bouts) were available to represent other gradients of life history, such as those of altricial/precocial homeotherms (Stearns 1983), periodic/equilibrium/opportunistic fish (Winemiller and Rose 1992), and bet-hedgers (Saether et al. 1996). For insects and other invertebrates, the slow–fast continuum has been investigated only in some hymenopterans and odonates (Blackburn 1991; Johansson 2000), and future studies should carefully consider tradeoffs between life-history traits operating from larval to adult stages.

The link between density feedback and the slow–fast continuum has been previously assessed in a few studies, albeit using contrasting metrics (i.e., density feedback shape and strength, population growth rate inflection points, process error, return rates), and from different population growth models such as theta-logistic variants (Saether et al. 2002, 2004; Saether and Engen 2002), age-structured autoregression (Lande et al. 2002, 2006; Saether et al. 2005), and polynomials (Fowler 1981, 1988; Sibly et al. 2007). Given these various choices, it is unclear to what extent results across those studies are comparable. Due to severe fitting issues with the theta-logistic model such as the inherent play-offs between the shape parameter *θ* and maximum rate of population increase *r*_*m*_ (Clark et al. 2010), correlations between *θ* and life history must be revisited. The assignment of clear biological meaning to model parameters would certainly facilitate understanding of the generality of results across taxa and studies. Of particular relevance to assessing correlations between long-term demographic data and life history, is the understanding of how measurement error affects estimates of density feedback (Freckleton et al. 2006; Knape and de Valpine 2012). We found that model rankings remained unchanged after the introduction of between 5% (all taxa and birds) and 10% (mammals) additive measurement error in simulated time series. Further work is required to specify, not only whether measurement error can affect feedback detection and parameter estimation by phenomenological models (Freckleton et al. 2006) but which error thresholds begin to erode the characteristics and biological interpretation of population growth curves.

## Conclusion

The mechanistic implication of our findings is that life history is correlated with a degree of measurable demographic variation by making species prone to experience larger or smaller negative crowding effects through trophic and social processes, regardless of stochastic forces. How and what processes relate to life history remains controversial, even for (the best-studied) mammals (Caughley and Krebs 1983; Krebs 2009). Increasing strength of density feedback can enhance population recovery, yet also magnify population variability in the smallest species, hence potentially making these species more vulnerable to extinction – an outcome of high predictive value for fishery collapses (Anderson et al. 2008). Recent emphasis on extinction dynamics caused by stochastic factors (Melbourne and Hastings 2008) should also take into account social/trophic interactions driving strong density feedback with extreme population variability (Brook et al. 2008). Methodologically, this underlines the need for broader application of models capturing eruptive dynamics in the analysis of long-term censuses, but for both slow (Forsyth and Caley 2006) and fast species.

Managers and conservationists can resort to generalized life-history estimates to predict population recoveries following harvesting and environmental shocks, and to rank species by the propensity to undergo particular patterns of change. In particular, our results are applicable where management and conservation priorities need to be made on the basis of rankings of species' conservation status (Knapp et al. 2003), in the absence of detailed demographic or, in general, quantitative data (Tulloch et al. 2011), such as in the monitoring of areas planned for development, exploitation, protection, or focal investment on population recovery (Possingham et al. 2002). The strength of density feedback indicates rebound capacity from low numbers, which can be approximated by the position of a species along the slow–fast continuum, and such approximation could complement other qualitative measures of conservation status attempting to optimize the allocation of always-limited resources (Possingham et al. 2002; Tulloch et al. 2011).

## Supporting Information

**Figure S1.** Standardized BIC-weighted effect sizes for four life-history traits (*Body size*, *Longevity*, *Fertility*) as predictors of variation in census-derived strength of compensatory density feedback (response), over all time series supported for Gompertz growth: (*i*) all taxa (a,b: aquatic invertebrates, amphibians, birds, fish, insects, mammals, plants, reptiles), and the subsets of (*ii*) mammal (c,d) and (*iii*) bird (e,f) species. Left panels (a,c,e) come from a model set controlling for number of generations monitored (*G*), and right panels (b,d,f) from a model set controlling for *G* and *Body size*. Bold lines represent *w*BIC medians as obtained from 100 bootstrapped samples. Fits were obtained using GLMM which accounted for phylogenetic nonindependence at the Linnaean taxonomical level of *Class*, and GLM for the subset analyses of mammals and birds. We show model sets in Table 1, sample sizes in Table S1, and BIC metrics in Table S4.

**Figure S2** Standardized BIC-weighted effect sizes for four life-history traits (*Body size*, *Longevity*, *Age at first reproduction*, *Fertility*) as predictors of variation in census-derived strength of compensatory density feedback (response), over high-quality time series supported for Gompertz growth: (*i*) all taxa (a,b: aquatic invertebrates, amphibians, birds, fish, insects, mammals, plants, reptiles), and the subsets of (*ii*) mammal (c,d) and (*iii*) bird (e,f) species. Left panels (a,c,e) come from a model set controlling for time-series length (*q*), and right panels (b,d,f) from a model set controlling for *q* and *Body size*. Bold lines represent *w*BIC medians as obtained from 100 bootstrapped samples. Fits were obtained using GLMM which accounted for phylogenetic nonindependence at the Linnean taxonomical level of *Class*, and GLM for the subset analyses of mammals and birds. We show model sets in Table 1, sample sizes in Table S1, and BIC metrics in Table S5.

**Figure S3.** Standardized BIC-weighted effect sizes for four life-history traits (*Body size*, *Longevity*, *Fertility*) as predictors of variation in census-derived strength of compensatory density feedback (response), high-quality time series supported for Gompertz growth: (*i*) all taxa (a,b: aquatic invertebrates, amphibians, birds, fish, insects, mammals, plants, reptiles), and the subsets of (*ii*) mammal (c,d) and (*iii*) bird (e,f) species. Left panels (a,c,e) come from a model set controlling for number of generations monitored (*G*), and right panels (b,d,f) from a model set controlling for *G* and *Body size*. Bold lines represent *w*BIC medians as obtained from 100 bootstrapped samples. Fits were obtained using GLMM which accounted for phylogenetic nonindependence at the Linnean taxonomical level of *Class*, and GLM for the subset analyses of mammals and birds. We show model sets in Table 1, sample sizes in Table S1, and BIC metrics in Table S6.

**Table S1.** Number of species analyzed after removing missing life-history traits, selecting only time series supported by Gompertz growth, and removing time series supported for depensatory density feedback. Time series showing Gompertz growth were split by core and redundant species (see Methods), and all analyses were replicated for all species' time series, and only high-quality times series (Table S2). Colored numbers in table match with colored text below relative to the four types of analyses undertaken.

**Table S2.** Summary of stages used to obtain the highquality time series with extreme departures from the assumptions of the Ricker and Gompertz growth models.

**Table S3.** Summary of content of figures and tables for model sets accounting for life-history effects on strength of compensatory density feedback. Control variables are time-series length (*q*) and number of generations monitored (*G* = *q*/age at first reproduction).

**Table S4.** Bayesian information criterion (BIC) support for the top-ranked models^a^ derived from GLMM relating life history to strength of compensatory density feedback for all time series supported for Gompertz growth, including all major groups (aquatic invertebrates, amphibians, birds, fish, insects, mammals, plants, reptiles), and the subsets of mammal and bird species. *w*BIC, %DE, and Δ%DE are medians (in bold) from 100 bootstrapped samples (95% confidence intervals)^b^. Models included number of generations monitored (*G*) and body size (*Body*) as controls. We show effect sizes in Figure S1, model sets in Table 1, and sample sizes in Table S1.

**Table S5.** Bayesian information criterion (BIC) support for the top-ranked models^a^ derived from generalized linear mixed modeling relating life history to strength of compensatory density feedback for high-quality time series supported for Gompertz growth, including all major groups (aquatic invertebrates, amphibians, birds, fish, insects, mammals, plants, reptiles), and the subsets of mammal and bird species. *w*BIC, %DE, and Δ%DE are medians (in bold) from 100 bootstrapped samples (95% confidence intervals)^b^. Models included time-series length (*q*) and body size (*Body*) as controls. We show effect sizes in Figure S2, model sets in Table 1, and sample sizes in Table S1.

**Table S6.** Bayesian information criterion (BIC) support for the top-ranked models^a^ derived from generalized linear mixed models relating life history to strength of compensatory density feedback for high-quality time series supported for Gompertz growth, including all major groups (aquatic invertebrates, amphibians, birds, fish, insects, mammals, plants, reptiles), and the subsets of mammal and bird species. *w*BIC, %DE and Δ%DE are medians (in bold) from 100 bootstrapped samples (95% confidence intervals)^b^. Models included number of generations monitored (*G*) and body size (*Body*) as controls. We show effect sizes in Figure S3, model sets in Table 1, and sample sizes in Table S1.

**Table S7.** Bayesian information criterion (BIC) support for the top-ranked models derived from GLMM (all taxa: aquatic invertebrates, amphibians, birds, fish, insects, mammals, plants, reptiles) and GLM (birds and mammals) relating life history to strength of compensatory density feedback for time series supported for Gompertz growth. *w*BIC, %DE, and Δ%DE are medians (in bold) from 100 bootstrapped samples (95% percentile ranges)^a^. Models included time-series length (*q*, years) and body size (*Body*, mm) as controls and three further life-history traits^b^, and we present results for observed (as in Table 2) and simulated (below) time series with incorporation of 5%, 10%, and 15% of measurement error. Samples sizes are given in Table S1.
