# Supplementary material for: Strength of density feedback in census data increases from slow to fast life histories
Source: Ecol Evol. 2012 Jul 12;2(8):1922–34. doi: 10.1002/ece3.298 (PMC3433995; doi:10.1002/ece3.298)
Supplement: Supplementary file 1 [file ece30002-1922-SD8.docx]

**SUPPORTING INFORMATION**

Sample sizes for all analyses undertaken

Page 2

**Selection of high-quality time series**

Pages 3 and 4

**Model fits for complementary analyses**

Pages 5 to 11

**Examination of measurement error**

Pages 12 and 13

**References**

Pages 14 and 15

Sample sizes for all analyses undertaken

**Table S1.** Number of species analysed after removing missing life-history traits, selecting only time series supported by Gompertz growth, and removing time series supported for depensatory density feedback. Time series showing Gompertz growth were split by core and redundant species (see Methods), and all analyses were replicated for all species’ time series, and only high-quality times series (Table S2). Coloured numbers in table match with coloured text below relative to the four types of analyses undertaken.

|  | **All  time series** | | **High-quality  time series** | |
| --- | --- | --- | --- | --- |
| **Total** | **1198^a^** | | **812^a^** | |
| *minus missing life history* | **1177^b^** | | **795^b^** | |
| Aquatic invertebrates | 36 | | 21 | |
| Birds | 225 | | 145 | |
| Fish | 109 | | 65 | |
| Herpetiles | 37 | | 17 | |
| Insects | 588 | | 476 | |
| Mammals | 152 | | 62 | |
| Plants | 30 | | 9 | |
|  | **Gompertz-growth supported** | | | |
|  | *Core species* | *Redundant species* | *Core species* | *Redundant species* |
| Total | 328 | 446 | 191 | 392 |
| *minus depensatory density feedback* | **326^c^** | **446^c^** | **190^c^** | **392^c^** |
| Aquatic invertebrates | 13 | 5 | 6 | 5 |
| **Birds** | **123^d^** | **26^d^** | **80^d^** | **24^d^** |
| Fish | 37 | 16 | 22 | 13 |
| Herpetiles | 20 | 0 | 13 | 0 |
| Insects | 38 | 390 | 20 | 350 |
| **Mammals** | **90^d^** | **0^d^** | **45^d^** | **0^d^** |
| Plants | 5 | 9 | 4 | 0 |

^a^ Single-species population models (Ricker-logistic and Gompertz, exponential and random walk) fitted to time series of population abundance

^b^ Principal component analysis based on correlations among the four life-history traits (Age at first reproduction, Body size, Fertility, Longevity)

^c^ GLMM relating strength of compensatory density feedback (response) to life-history traits (predictors) across all taxa [Taxonomic Class = random factor]

^d^ GLM relating strength of compensatory density feedback (response) to life-history traits (predictors) in birds and mammals

Selection of high-quality time series

We replicated model contrasts on a high-quality subset of time series after assessing low sample size, extreme non-stationarity, missing values and outliers, as follows (see summary in Table S2):

1. The statistical ‘detection’ of density feedback increases with time-series length ^1-3^, which also alters variability in parameters estimated in models of population dynamics ^4^; so we excluded the shortest 101 time series (i.e. < 14 time steps).
2. Stationarity implies that endogenous processes driving population dynamics, such as density feedback, vary around mean levels with constant variance ^5^. This property allows reliable reconstruction of those processes through density-dependent models ^5,6^. We assessed stationarity through the variability in ‘return rate’, i.e. “the time it takes [for a population] to return to equilibrium following disturbance” ^7^ in log*_e_*(*N*)-*t* space. Given a time series, a variance considerably smaller than a mean of the ‘return time’ is indicative of a relatively constant period of oscillation ^7, p. 99-100^. Thus, we excluded 140 time series with a coefficient of variation of the return rate > 3 (i.e. > 75% percentile). Furthermore, stationary time series should show no temporal trending in population size, so we further excluded 93 time series for which linear trending of log-transformed population sizes had more AIC*_c_*-support than an intercept-only model and slopes > |0.1| ( < 25% and > 75% percentiles).
3. The frequency of missing values in a time series was regarded a sign of poor quality, so we removed those 14 stationary time series where missing-value frequency was ≥ 20% of their length.
4. We detected time series with extreme outliers by studentizing the residuals ^8^ from the linear-trending model used above, and this resulted in a further exclusion of 38 time series with residuals > 3.4 (> 75% percentile).

For the high-quality time series, we report model rankings in Tables S4 to S6, and model-averaged fixed effects in Figures S1 to S3.

| Table S2. Summary of stages used to obtain the high-quality time series with extreme departures from the assumptions of the Ricker and Gompertz growth models. | | |
| --- | --- | --- |
| Criteria | Threshold | Time-series type removed  y axis = population size (*N*)  x axis = years |
| **Time-series length** | < 14 time steps |  |
| **Variation of return rates** | > 3 |  |
| **ln(*N*) trending** | slope > \|0.1\| |  |
| **Missing values** | > 20 % of data points |  |
| **Outliers** | studentized residual > 3.4 |  |

Model fits for complementary analyses

**Table S3.** Summary of content of figures and tables for model sets accounting for life-history effects on strength of compensatory density feedback. Control variables are time-series length (*q*) and number of generations monitored (*G* = *q*/age at first reproduction).

| **Control variable** | **All time series** | **High-quality time series** |
| --- | --- | --- |
| *q* | **Table 1**  **Figure 1**  **Table S7** (measurement error) | **Table S5**  **Figure S2** |
| *G* | **Table S4**  **Figure S1** | **Table S6**  **Figure S3** |

**Models included number of generations monitored (*G*) and Body size (*Body*) as controls, for all time series (Table S4, Figure S1)**

**Table S4.** Bayesian information criterion (BIC) support for the top-ranked models^a^ derived from GLMM relating life history to strength of compensatory density feedback for all time series supported for Gompertz growth, including all major groups (aquatic invertebrates, amphibians, birds, fish, insects, mammals, plants, reptiles), and the subsets of mammal and bird species. *w*BIC, %DE and Δ%DE are medians (in bold) from 100 bootstrapped samples (95% confidence intervals)^b^. We show effect sizes in Figure S1, model sets in Table 1, and sample sizes in Table S1.

| Data set | Control variable | Top-ranked model  per model set | *w*BIC | %DE | Δ%DE | ER | % Top-rank consistency |
| --- | --- | --- | --- | --- | --- | --- | --- |
| All taxa | *G* | *Strength ~ G+Long* | **1.00**  [0.00 to 1.00] | **20.6**  [13.1 to 31.7] | **20.5**  [12.8 to 30.0] | **> 1000** | **79**  (5) |
| All taxa | *G*,*Body* | *Strength ~ G+Body+Long* | **1.00**  [0.35 to 1.00] | **25.6**  [18.5 to 34.9] | **25.1**  [17.9 to 34.2] | **> 1000** | **96**  (4) |
| Mammals | *G* | *Strength ~ G+Body* | **0.87**  [0.19 to 0.90] | **43.0**  [25.3 to 61.0] | **34.0**  [12.2 to 51.2] | **> 1000** | **93**  (5) |
| Mammals | *G*,*Body* | *Strength ~ G+Body* | **0.72**  [0.15 to 0.81] | **43.0**  [25.3 to 61.0] | **34.0** [12.2 to 51.2] | **> 1000** | **89**  (9) |
| Birds | *G* | *Strength ~ G+Long* | **0.60**  [0.03 to 0.91] | **17.6**  [8.5 to 29.4] | **15.8**  [7.0 to 28.0] | **> 1000** | **66**  (27) |
| Birds | *G*,*Body* | *Strength ~ G+Body+Long* | **0.42**  [0.04 to 0.91] | **18.9**  [9.1 to 32.2] | **17.1**  [7.2 to 30.7] | **> 1000** | **56**  (38) |

**^a^ Model sets**: 1 single response [*Strength* of compensatory density feedback], *and* 1 or 2 life-history predictors [*Body* = Body size (mm), *Fert* = Fertility (number of young per year) and *Long* = Longevity (maximum age attained in the wild, months)], *and* 1 or 2 control variables [*G* = number of generations monitored, and *Body*].

**^b^ BIC metrics**: ***w*BIC =** BIC Model probabilities given each data and model set, %**DE** = % Deviance in *Strength* explained by each model within each model set, **Δ%DE** = % Deviance in *Strength* explained by each model minus % Deviance in *Strength* explained by the model including only *G* (i.e. Deviance in *Strength* explained by life history conditional on *q*), **ER** = Evidence ratio of the top-ranked model *w*BIC to only-*G* model *w*BIC within each model set (i.e. times support for top-ranked model equating life-history traits was larger than for only-*G*), and **%Top-ranked consistency** = times each model was top-ranked over the 100 bootstrapped samples (in brackets: times each model was not the top ranked model yet received considerable support [ΔBIC < 4]).


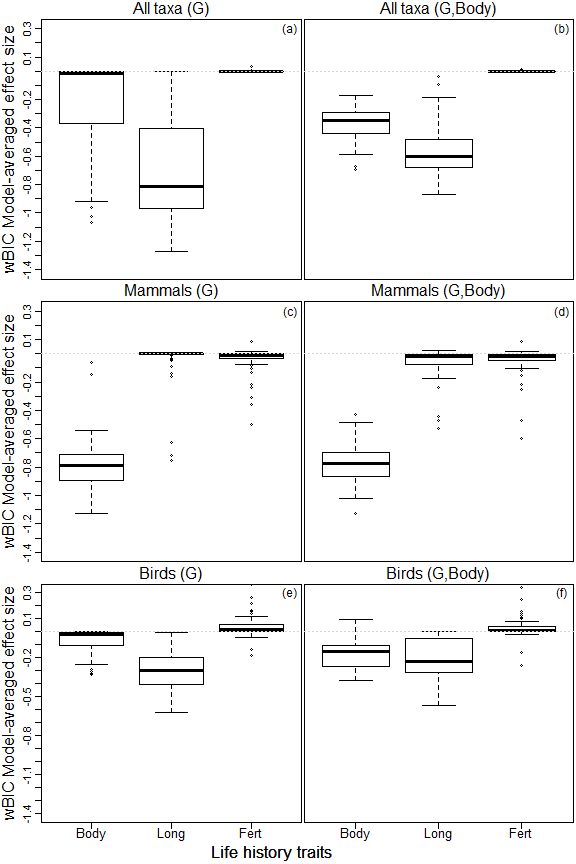


**Fig. S1.** Standardized BIC-weighted effect sizes for four life-history traits (*Body size*, *Longevity*, *Fertility*) as predictors of variation in census-derived strength of compensatory density feedback (response), over all time series supported for Gompertz growth: (*i*) all taxa (a,b: aquatic invertebrates, amphibians, birds, fish, insects, mammals, plants, reptiles), and the subsets of (*ii*) mammal (c,d) and (*iii*) bird (e,f) species. Left panels (a,c,e) come from a model set controlling for number of generations monitored (*G*), and right panels (b,d,f) from a model set controlling for *G* and *Body size*. Bold lines represent *w*BIC medians as obtained from 100 bootstrapped samples. Fits were obtained using GLMM which accounted for phylogenetic non-independence at the Linnaean taxonomical level of *Class*, and GLM for the subset analyses of mammals and birds. We show model sets in Table 1model support in Table S4. We show model sets in Table 1, sample sizes in Table S1, and BIC metrics in Table S4.

**Models included time series length (*q*) and body size (*Body*) as controls, for the high-quality dataset (Table S5, Figure S2)**

**Table S5.** Bayesian information criterion (BIC) support for the top-ranked models^a^ derived from generalized linear mixed modelling relating life history to strength of compensatory density feedback for high-quality time series supported for Gompertz growth, including all major groups (aquatic invertebrates, amphibians, birds, fish, insects, mammals, plants, reptiles), and the subsets of mammal and bird species. *w*BIC, %DE and Δ%DE are medians (in bold) from 100 bootstrapped samples (95% percentile confidence intervals)^b^. We show effect sizes in Figure S2, model sets in Table 1, and sample sizes in Table S1.

| Data set | Control variable | Top-ranked model  per model set | *w*BIC | %DE | Δ%DE | ER | % Top-rank consistency |
| --- | --- | --- | --- | --- | --- | --- | --- |
| All taxa | *q* | *Strength ~ q+Long* | **0.83**  [0.00 to 1.00] | **27.2**  [16.9 to 39.5] | **16.5**  [5.8 to 27.5] | **> 1000** | **67** (15) |
| All taxa | *q*,*Body* | *Strength ~ q+Body* | **0.55**  [0.00 to 0.98] | **25.8**  [15.5 to 37.5] | **15.5**  [4.4 to 26.6] | **> 1000** | **55**  (22) |
| Mammals | *q* | *Strength ~ q+Long* | **0.28**  [0.00 to 0.85] | **39.8**  [22.3 to 67.0] | **30.5**  [12.9 to 61.1] | **> 1000** | **41**  (41) |
| Mammals | *q*,*Body* | *Strength ~ q+Body* | **0.41**  [0.00 to 0.0.63] | **39.8**  [22.3 to 67.0] | **30.5**  [12.9 to 61.1] | **> 1000** | **65**  (28) |
| Birds | *q* | *Strength ~ q+Long* | **0.61**  [0.02 to 0.89] | **20.8**  [8.1 to 38.1] | **14.4**  [2.3 to 27.8] | **> 100** | **72**  (18) |
| Birds | *q*,*Body* | *Strength ~ q+Body+Long* | **0.31**  [0.0 to 0.89] | **21.8**  [9.6 to 39.7] | **16.0**  [4.5 to 27.8] | **19** | **50**  (30) |

**^a^ Model sets**: 1 single response [*Strength* of compensatory density feedback, *and* 1 or 2 life-history predictors [*Age* = Age at first reproduction (months), *Body* = Body size (mm), *Fert* = Fertility (number of young per year) and *Long* = Longevity (maximum age attained in the wild, months)].

**^b^ BIC metrics**: ***w*BIC** = BIC Model probabilities given each data and model set, %**DE** = % Deviance in *Strength* explained by each model within each model set, **Δ%DE** = % Deviance in *Strength* explained by each model minus % Deviance in *Strength* explained by the model including only *q* (i.e. Deviance in *Strength* explained by life history conditional on *q*), **ER** = Evidence ratio of the top-ranked model *w*BIC compared to *q*-only model *w*BIC within each model set (i.e. times support for top-ranked model equating life-history traits was larger than for the only-*q* model), and **%Top-ranked consistency** = times each model was top-ranked over the 100 bootstrapped samples (in brackets: times each model was not the top ranked model yet received considerable support [ΔBIC < 4]).

**
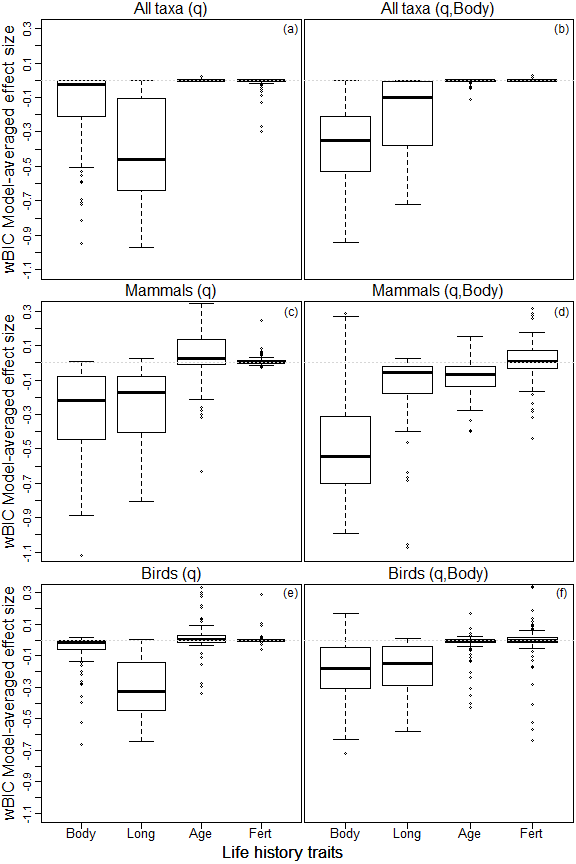
**

**Fig. S2** Standardized BIC-weighted effect sizes for four life-history traits (*Body size*, *Longevity*, *Age at first reproduction*, *Fertility*) as predictors of variation in census-derived strength of compensatory density feedback (response), over high-quality time series supported for Gompertz growth: (*i*) all taxa (a,b: aquatic invertebrates, amphibians, birds, fish, insects, mammals, plants, reptiles), and the subsets of (*ii*) mammal (c,d) and (*iii*) bird (e,f) species. Left panels (a,c,e) come from a model set controlling for time-series length (*q*), and right panels (b,d,f) from a model set controlling for *q* and *Body size*. Bold lines represent *w*BIC medians as obtained from 100 bootstrapped samples. Fits were obtained using GLMM which accounted for phylogenetic non-independence at the Linnean taxonomical level of *Class*, and GLM for the subset analyses of mammals and birds. We show model sets in Table 1, sample sizes in Table S1, and BIC metrics in Table S5.

**Models included number of generations monitored (*G*) and Body size (*Body*) as controls, for high-quality dataset (Table S6, Figure S3)**

**Table S6.** Bayesian information criterion (BIC) support for the top-ranked models^a^ derived from generalized linear mixed models relating life history to strength of compensatory density feedback for high-quality time series supported for Gompertz growth, including all major groups (aquatic invertebrates, amphibians, birds, fish, insects, mammals, plants, reptiles), and the subsets of mammal and bird species. *w*BIC, %DE and Δ%DE are medians (in bold) from 100 bootstrapped samples (95% confidence intervals)^b^. We show effect sizes in Figure S3, model sets in Table 1, and sample sizes in Table S1.

| Data set | Control variable | Top-ranked model  per model set | *w*BIC | %DE | Δ%DE | ER | % Top-rank consistency |
| --- | --- | --- | --- | --- | --- | --- | --- |
| All taxa | *G* | Strength ~ *G*+Long | **0.98**  [0.01 to 1.00] | **23.1**  [11.8 to 34.4] | **20.0**  [10.2 to 32.4] | **> 1000** | **75**  (7) |
| All taxa | *G*,*Body* | *Strength ~ G+Body* | **0.55**  [0.21 to 0.98] | **25.8**  [15.5 to 37.5] | **15.5**  [4.4 to 26.6] | **> 1000** | **55**  (22) |
| Mammals | *G* | *Strength ~ G+Long* | **0.35**  [0.00 to 0.84] | **40.6**  [21.6 to 63.5] | **24.2**  [7.9 to 42.3] | **> 1000** | **50**  (25) |
| Mammals | *G*,*Body* | *Strength ~ G+Body* | **0.37**  [0.01 to 0.73] | **38.3**  [21.0 to 66.61] | **24.6** [8.5 to 40.4] | **> 1000** | **49**  (35) |
| Birds | *G* | *Strength ~ G+Long* | **0.62**  [0.01 to 0.89] | **21.8**  [10.5 to 37.6] | **19.0**  [6.3 to 33.7] | **> 900** | **74**  (15) |
| Birds | *G*,*Body* | *Strength ~ G+Body+Long* | **0.47**  [0.02 to 0.88] | **23.5**  [11.6 to 38.5] | **20.5**  [8.8 to 34.1] | **> 200** | **58**  (28) |

**^a^ Model sets**: 1 single response [*Strength* of compensatory density feedback, *and* 1 or 2 life-history predictors [*Body* = Body size (mm), *Fert* = Fertility (number of young per year) and *Long* = Longevity (maximum age attained in the wild, months)], *and* 1 or 2 control variables [*G* = number of generations monitored, and *Body*].

**^b^ BIC metrics**: ***w*BIC =** BIC Model probabilities given each data and model set, %**DE** = % Deviance in *Strength* explained by each model within each model set, **Δ%DE** = % Deviance in *Strength* explained by each model minus % Deviance in *Strength* explained by the model including only *G* (i.e. Deviance in *Strength* explained by life history conditional on *q*), **ER** = Evidence ratio of the top-ranked model *w*BIC to only-*G* model *w*BIC within each model set (i.e. times support for top-ranked model equating life-history traits was larger than for only-*G*), and **%Top-ranked consistency** = times each model was top-ranked over the 100 bootstrapped samples (in brackets: times each model was not the top ranked model yet received considerable support [ΔBIC < 4]).

**
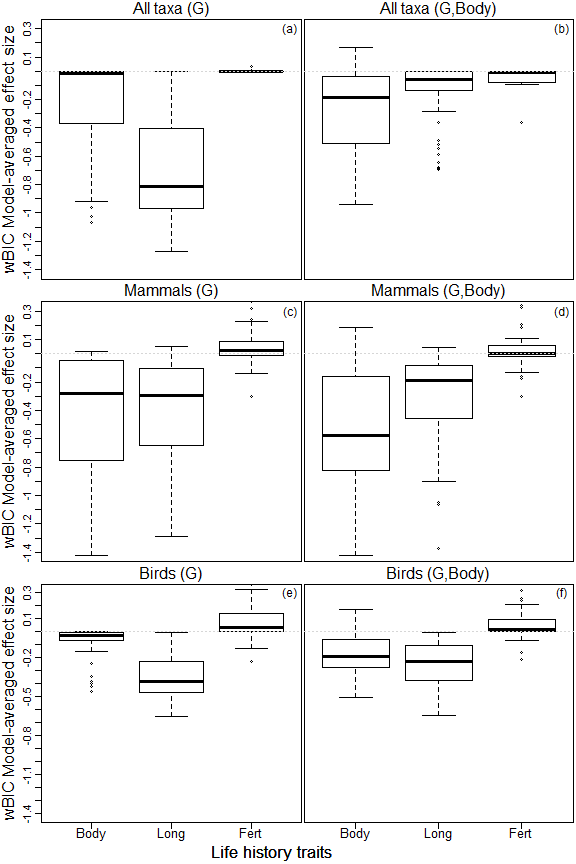
**

**Fig. S3.** Standardized BIC-weighted effect sizes for four life-history traits (*Body size*, *Longevity*, *Fertility*) as predictors of variation in census-derived strength of compensatory density feedback (response), high-quality time series supported for Gompertz growth: (*i*) all taxa (a,b: aquatic invertebrates, amphibians, birds, fish, insects, mammals, plants, reptiles), and the subsets of (*ii*) mammal (c,d) and (*iii*) bird (e,f) species. Left panels (a,c,e) come from a model set controlling for number of generations monitored (*G*), and right panels (b,d,f) from a model set controlling for *G* and *Body size*. Bold lines represent *w*BIC medians as obtained from 100 bootstrapped samples. Fits were obtained using GLMM which accounted for phylogenetic non-independence at the Linnean taxonomical level of *Class*, and GLM for the subset analyses of mammals and birds. We show model sets in Table 1, sample sizes in Table S1, and BIC metrics in Table S6.

Examination of measurement error

We examined the potential effect of measurement error in our correlates of density feedback and life history in four stages. First, we simulated 772 times series using the parameters (intercept, slope, sigma, carrying capacity) for the 772 time series supported for Gompertz growth (Table S1: 326 core + 446 redundant species). Second, we introduced measurement error in each observation of each time series ⎯ to do so, we summed up the raw (simulated) value of each observation and a value obtained randomly from a normal distribution (*q*, *σ*), where *q* = length of time series and *σ* = measurement error/100. We repeated the simulation with three values of relatively high measurement error in census data: 5, 10 and 15 %. Third, we fitted the Gompertz model to the simulated time series with added measurement error. Lastly, we contrasted the models in our set (i.e., strength of density feedback as function of life history with controls for *q* and for body size, see models in Table 1) through GLMM (all taxa) and GLM (mammals and birds) and using strength of density feedback from simulated time series with each of the three magnitudes of added measurement error.

**Results**

Top-ranked models (highest *w*AIC*_c_* in the model set) remained the same and the deviance in strength of compensatory density feedback explained by life history was of the same magnitude for the original and simulated time series for 5 % measurement error in all taxa, mammals and birds, and for 10 % measurement error with a relative reduction in explained deviance only in mammals (Table S7). For the same dataset, Brook and Bradshaw (2006) had found that the most variable time series (potentially having the highest measurement errors) tended to have more information-theoretic support for density feedback yet contributed < 3% to underlying variation in parameter estimates across species.

**Table S7.** Bayesian information criterion (BIC) support for the top-ranked models derived from GLMM (all taxa: aquatic invertebrates, amphibians, birds, fish, insects, mammals, plants, reptiles) and GLM (birds and mammals) relating life history to strength of compensatory density feedback for time series supported for Gompertz growth. *w*BIC, %DE and Δ%DE are medians (in bold) from 100 bootstrapped samples (95% percentile ranges)^a^. Models included time series length (*q*, years) and body size (*Body*, mm) as controls and three further life history traits^b^, and we present results for observed (as in Table 2) and simulated (below) time series with incorporation of 5 %, 10 % and 15 % of measurement error. Samples sizes are given in Table S1.

| **Measurement error** | **Data set** | **Control** | **Top-ranked model** | ***w*BIC** | **%DE** | **Δ%DE** | **% Top-rank consistency** |
| --- | --- | --- | --- | --- | --- | --- | --- |
| Observed time series | All taxa | *q* | *Strength ~ q+Long* | **0.57** | **30.7** | **9.5** | **54** |
| 5 % | All taxa | *q* | *Strength ~ q+Long* | **0.95** | **22.4** | **4.1** | **78** |
| 10 % | All taxa | *q* | *Strength ~ q+Age* | **0.90** | **22.1** | **3.3** | **75** |
| 15 % | All taxa | *q* | *Strength ~ q* | **0.73** | **14.7** | **-** | **66** |
| Observed time series | All taxa | *q*,*Body* | *Strength ~ q+Body+Long* | **0.64** | **29.0** | **8.2** | **63** |
| 5 % | All taxa | *q*,*Body* | *Strength ~ q+Body+Long* | **0.69** | **22.4** | **7.8** | **65** |
| 10 % | All taxa | *q*,*Body* | *Strength ~ q+Body* | **0.38** | **21.1** | **2.4** | **47** |
| 15 % | All taxa | *q*,*Body* | *Strength ~ q* | **0.97** | **14.7** | **-** | **81** |
| Observed time series | Mammals | *q* | *Strength ~ q+Body* | **0.79** | **45.2** | **28.9** | **85** |
| 5 % | Mammals | *q* | *Strength ~ q+Body* | **0.67** | **40.3** | **14.1** | **79** |
| 10 % | Mammals | *q* | *Strength ~ q+Body* | **0.24** | **38.8** | **3.3** | **35** |
| 15 % | Mammals | *q* | *Strength ~ q* | **0.23** | **21.3** | **-** | **40** |
| Observed time series | Mammals | *q*,*Body* | *Strength ~ q+Body* | **0.59** | **45.2** | **28.3** | **80** |
| 5 % | Mammals | *q*,*Body* | *Strength ~ q+Body* | **0.61** | **40.3** | **14.1** | **74** |
| 10 % | Mammals | *q*,*Body* | *Strength ~ q+Body* | **0.27** | **38.8** | **3.3** | **39** |
| 15 % | Mammals | *q*,*Body* | *Strength ~ q* | **0.34** | **21.3** | **-** | **52** |
| Observed time series | Birds | *q* | *Strength ~ q+Long* | **0.60** | **19.4** | **10.0** | **75** |
| 5 % | Birds | *q* | *Strength ~ q+Long* | **0.67** | **18.7** | **8.7** | **72** |
| 10 % | Birds | *q* | *Strength ~ q* | **0.24** | **10.1** | **-** | **42** |
| 15 % | Birds | *q* | *Strength ~ q+Body* | **0.25** | **9.8** | **3.9** | **40** |
| Observed time series | Birds | *q*,*Body* | *Strength ~ q+Body+Long* | **0.29** | **21.1** | **11.0** | **43** |
| 5 % | Birds | *q*,*Body* | *Strength ~ q+Body+Long* | **0.32** | **19.7** | **9.0** | **47** |
| 10 % | Birds | *q*,*Body* | *Strength ~ q* | **0.44** | **10.1** | **-** | **58** |
| 15 % | Birds | *q*,*Body* | *Strength ~ q+Body* | **0.31** | **9.8** | **3.9** | **44** |

**^a^ *w*BIC =** BIC Model probabilities given each data and model set, %**DE** = % Deviance in *Strength* explained by each model within each model set, **Δ%DE** = % Deviance in *Strength* explained by each model minus % Deviance in *Strength* explained by the model including only *q*, and **%Top-ranked consistency** = times each model was top-ranked over the 100 bootstrapped sample.

**^b^ Traits:** *Age* = Age at first reproduction (years), *Body* = Body size (mm), *Fert* = Fertility (young/year) , *Long* = Longevity (years)

References

1 Woiwod, I. P. & Hanski, I. Patterns of density dependence in moths and aphids. *Journal of Animal Ecology* **61**, 619-629 (1992).

2 Wolda, H. & Dennis, B. Density dependence tests, are they? *Oecologia* **95**, 581-591 (1993).

3 Holyoak, M. & Lawton, J. H. Comment arising from a paper by Wolda and Dennis: using and interpreting the results of tests for density dependence. *Oecologia* **95**, 592-594 (1993).

4 Dennis, B., Ponciano, J. M., Lele, S. R., Taper, M. L. & Staples, D. F. Estimating density dependence, process noise, and observation error. *Ecological Monographs* **76**, 323-341 (2006).

5 Turchin, P. & Taylor, A. D. Complex dynamics in ecological time series. *Ecology* **73**, 289-305 (1992).

6 Royama, T. *Analytical population dynamics*. (Chapman & Hall, 1992).

7 Berryman, A. A. *Principles of population dynamics and their application*. (Stanley Thorners Ltd., 1999).

8 Cook, R. D. *Residuals and influence in regression*. (Chapman & Hall, 1982).
